# Genotypic characterization and comparison of Streptococcus mutans in American Indian and Southeast Iowa children

**DOI:** 10.1002/cre2.94

**Published:** 2017-12-22

**Authors:** Alissa L. Villhauer, David J. Lynch, John J. Warren, Deborah V. Dawson, Derek R. Blanchette, David R. Drake

**Affiliations:** ^1^ Iowa Institute for Oral Health Research University of Iowa, College of Dentistry Iowa USA; ^2^ Preventive and Community Dentistry University of Iowa, College of Dentistry Iowa USA

**Keywords:** genotypic diversity, genotypes, oral microbiology, early childhood caries, etiologic agents

## Abstract

Early childhood caries is a complex health care issue that has a multifactorial etiology. One aspect of this etiology is the colonization and propagation of acidogenic bacteria at an early age. There have been several bacterial species associated with caries but 1 common species is Streptococcus mutans. Here, we describe genotypic diversity and commonality of Streptococcus mutans recovered from children representing 2 groups with similar socioeconomic demographics: a Northern Plains American Indian Tribe and a Southeast Iowa population. Forty 36‐month‐old American Indian children were selected from a cohort of 239 mothers and children, and forty 2‐ to 5‐year‐old children from Southeast Iowa were selected to compare the genotypic profiles of Streptococcus mutans recovered from each child's plaque. S. mutans isolates were selected from whole mouth plaque samples; DNA was extracted and amplified via AP‐PCR to show specific genotype patterns. These patterns were compared with GelComparIIv6.5 gel analysis software. We found 18 distinct genotypes from 524 isolates; 13 of which were common between the 2 communities. Five genotypes were unique to only the American Indian children while the Southeast Iowa children harbored no unique genotypes. Although the American Indian children had some genotypes that were not present in the Southeast Iowa children, these were not widely distributed among the community. Furthermore, the levels of genotypic diversity and commonality were similar between the 2 populations. This study sets the groundwork for a comprehensive comparison of genotypes and caries among larger subsections of both populations.

## 1 INTRODUCTION

Early childhood caries (ECC) is a significant health problem, particularly in children living in low socioeconomic conditions (Congiu, Campus, & Luglie, 2014; Finlayson, Siefert, Ismail, & Sohn, 2007; Psoter, Pendrys, Morse, Zhang, & Mayne, 2006; Sankeshwari, Ankola, Tangade, & Hebbal, 2013; Schroth, Halchuk, & Star, 2013). Severe ECC (SECC) is a particularly aggressive form of caries with rapid demineralization and concomitant cavitation in very young children (Edelstein, Chinn, & Laughlin, 2009). As with most diseases, the factors contributing to the development of this disease are varied and complex, including components of the socioeconomic status (SES), education level of parents, diet and access to healthy foods, and of course, development of an oral microbial community of organisms that exert a high cariogenic effect (American Academy of Pediatrics CoNACHCPSFNI & Metis, 2011; Caufield, Li, & Bromage, 2012; Feldens, Giugliani, Vigo, & Vitolo, 2010; Hughes, Dahlan, Papadopolou, et al., 2012; Irvine, Holve, Krol, & Schroth, 2011; Kanasi, Dewhirst, Chalmers, et al., 2010; Palmer, Kent, Loo, et al., 2010; Palmer, Nielsen, Peirano, et al., 2012; Palmer, Vo, Hiles, et al., 2012; Plutzer & Keirse, 2012; Qin, Li, Zhang, & Ma, 2008; Saxena et al., 2008; Schroth, Jeal, Kliewer, & Sellers, 2012; Tanner, Kent, Holgerson, et al., 2011; Vadiakas, 2008). Many studies have explored ways to prevent the development of this disease including health education of parents, application of fluoride varnish in children in Head Start programs, and even treatment of mothers with chlorhexidine varnishes to prevent transmission of cariogenic bacteria to their children (Batliner, Fehringer, Tiwari, et al., 2014; Milgrom, Huebner, Mancl, Garson, & Grembowski, 2013; Robertson et al., 2013; Vachirarojpisan, Shinada, & Kawaguchi, 2005; Weintraub, Ramos‐Gomez, Jue, et al., 2006). Overall, the impact of these intervention strategies on ECC and SECC in populations such as the American Indians (AIs) has been nominal at best and many strategies have had no significant impact at all (Albino, Batliner, & Tiwari, 2017; Flores & Lin, 2013; Phipps, Ricks, Manz, & Blahut, 2012; Ricks, Phipps, & Bruerd, 2015).

Although the development of caries in children is dependent on many variables, as stated previously, colonization of cariogenic bacteria within the oral cavities of these children appears to occur at a very young age (Hughes et al., 2012; Palmer et al., 2010; Parisotto et al., 2010; Tanner et al., 2011; Tanner, Mathney, Kent, et al., 2011). This can lead to establishment of a plaque community dominated by organisms that drive the caries process. Although prevention and treatment of early childhood caries will involve intervention at many different levels, it is important to understand the development of the cariogenic oral microbial flora. As with any infectious disease, a better understanding of the microorganisms involved will help advance practices in prevention development.

Caries has a complex etiology involving many variables. A number of organisms have been implicated as playing a role in caries development and the establishment of a cariogenic microflora (Berkowitz, 2003; Irigoyen Camacho, Sanchez Perez, Garcia Perez, & Zepeda Zepeda, 2009; Kanasi et al., 2010; Misra, Tahmassebi, & Brosnan, 2007; Tanner et al., 2011). However, a primary role in caries development has been firmly established over the years for the mutans streptococci, and in most populations, *Streptococcus mutans* (Al Shukairy, Alamoudi, Farsi, Al Mushayt, & Masoud, 2006; Baca et al., 2012; Banas, 2004; Berkowitz, 1976; Berkowitz, 1985; Berkowitz & Jones, 1985; Berkowitz, Turner, & Green, 1980; Berkowitz, Turner, & Green, 1981; Carletto Korber, Cornejo, & Gimenez, 2005; Carletto‐Korber, Gonzalez‐Ittig, Jimenez, & Cornejo, 2010; Carver, 1982; Davey & Rogers, 1984; Germaine, 1984; Okada et al., 2012; Zhou et al., 2013). Therefore, our studies with AI children to date have focused on the mutans streptococci within the oral microbiome (Lynch, Villhauer, Warren, et al., 2015).

A number of studies have now shown considerable diversity in *S*. *mutans* genotypes (GTs) associated with both caries‐free and caries‐active children (Baca et al., 2008; Carletto‐Korber et al., 2010; Cheon, Moser, Wiener, et al., 2013; Jiang et al., 2012; Klein, Bang, Florio, et al., 2006; Lapirattanakul et al., 2008; Lembo, Longo, Ota‐Tsuzuki, Rodrigues, & Mayer, 2007; Liu, Zou, Shang, & Zhou, 2007; Lynch et al., 2015; Moser, Mitchell, Ruby, et al., 2010; Nakano, Lapirattanakul, Nomura, et al., 2007; Napimoga, Höfling, Klein, Kamiya, & Gonçalves, 2005; Pieralisi, Rodrigues, Segura, et al., 2009; Waterhouse, Swan, & Russell, 2007; Zhou, Qin, Qin, & Ge, 2011). Little is known about differences in *S*. *mutans* GTs across different populations, due to the difficulty of comparing results not produced in the same study and done by different methodologies.

Two different studies on early childhood caries and specific GTs of the cariogenic bacterium, *S*. *mutans*, have been conducted, one in an Iowa cohort and one in a Northern Plains American Indian Tribe. Diversity of *S*. *mutans* GTs in AI children has been shown by arbitrarily primed polymerase chain reaction (AP‐PCR; Lynch et al., 2015). Although each population has a high level of early childhood caries [46% of Southeast Iowa (SEI) children, 80% of AI children] and a similar percentage of families categorized as low SES (55% SEI, 49% AI), both the living conditions and overall environment are quite different. Therefore, the purpose of this study was to determine similarities and differences in *S*. *mutans* GTs in children with early childhood caries across two different populations from two studies that have been conducted within the same laboratory utilizing the same AP‐PCR protocol.

## 2 METHODS

### 2.1 Study populations and recruitment

Study population 1 (AI) was composed of 239 mothers who were pregnant or who had just given birth were recruited from a Northern Plains American Indian Tribe. All onsite research team members were AI and were under the guidance of a study director who was a senior dental hygienist in the tribe. Whole mouth plaque samples were collected periodically at eight visits from birth until 36 months of age.

Study population 2 (SEI) was composed of one hundred ninety 2‐ to 5‐year‐old children (mean = 3.6 years) were recruited from the University of Iowa Muscatine Pediatric Dental Clinic. The majority of the children were either Hispanic (~45%) or Caucasian. A half‐time study coordinator, who had experience in coordinating studies involving children, traveled to the dental clinic to recruit subjects in person. A small number of the subjects were recruited through Muscatine Head Start.

This investigation was focused on the comparison of genotypic profiles of *S*. *mutans* in 40 children from each population. Since the average age of the children in the Iowa population was 3.6 years, in order to have the closest equivalent in age when samples were collected, the *S*. *mutans* isolates from the 36‐month samples from the AI children were analyzed. SEI children were chosen based on the criteria that each child had at least 2 *S*. *mutans* isolates recovered. There were 40 SEI children with at least 2 *S*. *mutans* isolates. The 40 AI children in this study were randomly chosen from those who were *S*. *mutans* positive at 36 months out of the 239 children in the original birth cohort.

### 2.2 Consent

For the Northern Plains tribe, the Internal Review Board (IRB) on record was the Aberdeen Area IRB and approval was obtained. The study proposal was also presented to and received approval from the Tribal Research Review Board. For both study populations, approval was obtained from the University of Iowa IRB.

### 2.3 Clinical examination

For the AI children, the clinical examination process has been detailed in Warren et al (2012). Briefly, caries examinations used dmfs criteria adapted from those used by NHANES (Dye, Tan, Smith, et al., 2007) and were conducted by trained and calibrated dental hygienist‐examiners. These examinations were completed using the knee‐to‐knee method (Nowak & Warren, 2000). For the SEI children, examinations were conducted by one of the three trained and calibrated examiners using portable dental equipment. The study utilized the d1d2‐3 caries criteria developed by Warren et al. (2015). With both study populations, examinations were conducted using a halogen examination light and a DenLite® illuminated mirror (Integra Miltex, York, PA). Teeth were dried with either gauze (AI children) or compressed air SEI children) and dental explorers were used to remove debris and to confirm areas of suspected decay.

### 2.4 Collection of plaque samples

#### 2.4.1 AI samples

Plaque samples were collected by a trained and calibrated dental hygienist from AI children in their homes. Samples were collected at ≤30 days (baseline), 4, 8, 12, 16, 22, 29, and 36 months (±30 days). Whole mouth plaque samples were collected by swabbing all smooth surfaces of the teeth (or oral mucosa and tongues in infants without erupted teeth) with a sterile cotton swab. Swabs were placed into tubes containing Tryptic Soy Broth‐Yeast Extract (TSB‐YE; Difco, Sparks, MD, USA) with 10% glycerol. Collection tubes were refrigerated until shipment to the microbiology laboratory for processing. Samples were shipped via FedEX overnight to the microbiology laboratories in the Iowa Institute for Oral Health Research at the College of Dentistry, University of Iowa. Temperature was maintained by shipping samples in temperature‐controlled Saf‐T‐Temp™ packaging (Saf‐T‐Pak, Hanover, MD, USA).

#### 2.4.2 SEI samples

Plaque samples were collected using a gentle, noninvasive swabbing technique at the clinic prior to the dental examination. A sterile cotton swab was wiped over all smooth surfaces of the teeth. Swabs were placed into tubes containing reduced transport fluid and transported to the microbiology laboratories on ice.

### 2.5 Sample processing and isolation of S. mutans

Swab samples were vortexed for 3 min and then placed in a sonicating water bath for 1 min to provide homogeneous suspensions of plaque bacteria. The resulting suspensions were diluted and plated on mitis‐salivarius‐kanamycin‐bacitracin (MSKB) agar (Difco, Sparks, MD, USA) using an Autoplate® Spiral Plating System (Advanced Instruments, Inc., Norwood, MA, USA) for determination of *S*. *mutans* counts. Plates were incubated at 37 °C, 5% CO_2_ for 72–96 hr.

Ten colonies displaying typical *S*. *mutans* colony morphology were selected from the mitis‐salivarius‐kanamycin‐bacitracin plate. Isolates were identified by fermentation profile (mannitol, raffinose, salicin, and sorbitol) and arginine decarboxylase activity. If there were fewer than 10 colonies, then all available colonies were selected. The average number of isolates obtained per subject in the SEI and AI cohorts was 4.65 and 8.17, respectively. In the AI population, a significant amount of *Streptococcus sobrinus* was present as well (approximately 30% of isolates). Isolates from this population were identified via fermentation profile or a second protocol which included preliminary identification by colony morphology and confirmation of species identification via PCR using primers specific to the *gtfB* (*S*. *mutans*) and the *gtfI* (*S*. *sobrinus*) genes (Villhauer, Lynch, & Drake, 2017). Following a second, single colony isolation onto TSB‐YE agar (48 hr, 37 °C, 5% CO_2_) to assure strain purity, isolates were frozen in TSB‐YE (10% glycerol) and stored at −80 °C.

### 2.6 DNA extraction and AP‐PCR

Isolates were cultured in TSB‐YE for 24 hr at 37 °C, 5% CO_2_. DNA was extracted using the Epicentre® MasterPure™ Gram Positive DNA Purification Kit (Epicentre, Madison, WI, USA) with the following modifications: (a) 25‐ml culture resuspended in 1.8‐ml TE, (b) 2‐μl Ready‐Lyse with 1‐hr incubation, (c) 25‐min Proteinase K incubation, (d) 1‐hr RNase A incubation after Proteinase K incubation, and (e) sample divided into three tubes for DNA precipitation steps. Due to the high volumes of mutans streptococci isolates being processed as part of the AI study, a new identification scheme, including a more rapid DNA extraction protocol, was tested and adopted as detailed by Villhauer et al (2017). Although both methods of DNA extraction were utilized within these subject sets, the majority of isolates were processed using the rapid DNA extraction protocol. Genotypic diversity was examined by AP‐PCR using the primer OPA‐2 (5′‐TGCCGAGCTG‐3′). Each 50‐μl PCR reaction contained 2‐μl template DNA (50 ng/μl), 5 μl of 10X PCR buffer, 200 μM of dNTP, 7 mM MgCl_2_, 2.5 U Taq polymerase, and 4 μm of OPA‐2 primer. *S*. *mutans* ATCC 25175 was used as a positive control for all reactions. Amplification was performed in a thermocycler (Eppendorf, Hauppauge, NY) programmed with the following temperature profile: initially 5 min at 94 °C, followed by 45 cycles of denaturation at 94 °C for 1 min, annealing at 36 °C for 1 min, and elongation at 72 °C for 2 min. Amplified products were electrophoresed on a 1.5% agarose gel and stained with ethidium bromide. A 100bp DNA ladder served as a molecular size marker on the gels. Gel images were captured using a transilluminator and digital imaging system (Fotodyne, Hartland, WI, USA).

### 2.7 Diversity analyses

Genotypic diversity was assessed by generation of dendrograms using GelCompar**®**IIv6.5 software (Applied Maths, Austin, TX, USA). Curve based cluster analysis (1% optimization, 1% curve smoothing) using the Pearson correlation and unweighted pair group method using arithmetic averages was used to assess strain relatedness. *S*. *mutans* isolates displaying greater than 70% similarity were considered to be the same GT (Damle, Yadav, Garg, et al., 2016; Mitchell, Ruby, Moser, et al., 2009; Moser et al., 2010).

## 3 RESULTS

### 3.1 Analysis of S. mutans GT profiles

**Figure 1:**
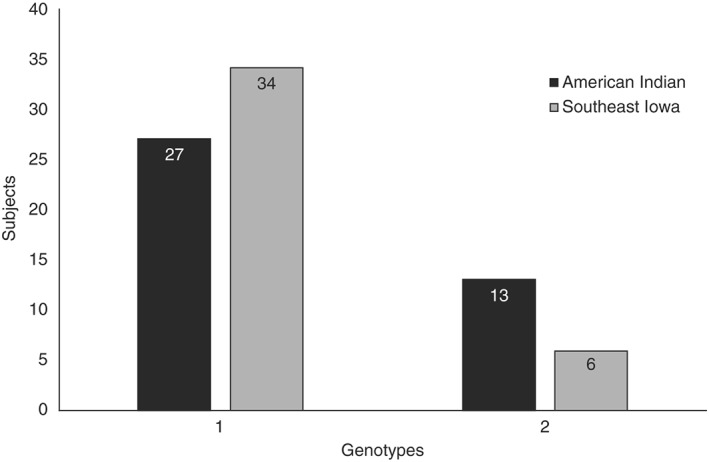
Number of genotypes per subject broken down by population. All children had either one or two genotypes (denoted on the X axis). The number of children (subjects) are shown on the Y axis with American Indian children represented by the black bars and Iowa children represented by grey bars

Five hundred twenty‐four isolates were analyzed (AI = 348; SEI = 176). A range of one to two GTs per person was seen in both study populations (Figure 1), with the AI children displaying an average of 1.33 GT per subject compared to 1.15 in the SEI children. The average for the entire subject set was 1.24. AI children had a higher percentage of two GTs than the Iowa children: 32.5% and 15%, respectively.

**Figure 2:**
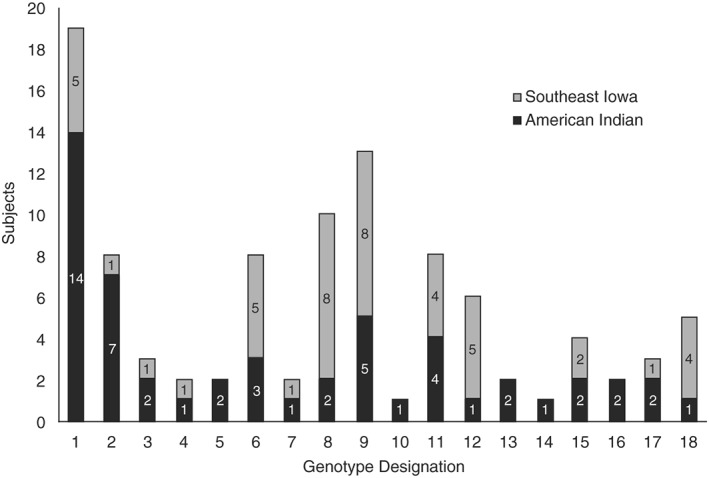
Distribution of genotypes within the study populations. Each unique genotype was assigned a number (1‐18; X axis) and the number of individuals (Y axis) who harbored each particular genotype is represented by the black (American Indian) and/or grey (Southeast Iowa) stacked bars

**Figure 3:**
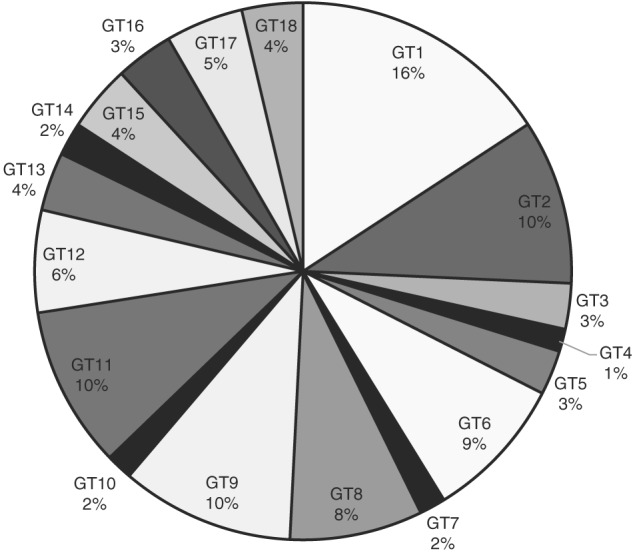
Percentage of total isolates representing each genotype (GT). GT1–GT18 are shown in this pie chart based on their representation of total isolates regardless of their population

Eighteen distinct *S*. *mutans* GTs were found in these 80 children (Figure 2). Thirteen of the GTs were detected in both study populations. Five GTs were found to be unique to the AI children, while none were unique to the SEI children. Some GTs were more prevalent in one population than the other (Table 1). In the AI children, GT1 is the most common and was detected in 35% of the subjects. GT8 and GT9 were the most common in the SEI children and were both found in 20% of those subjects. Five hundred twenty‐four *S*. *mutans* isolates were analyzed from the subjects. The most commonly isolated was GT1, which accounted for 16% of the total isolates, followed by GT2, GT9, and GT11 (each at 10% of total isolates; Figure 3).

**Table 1:**
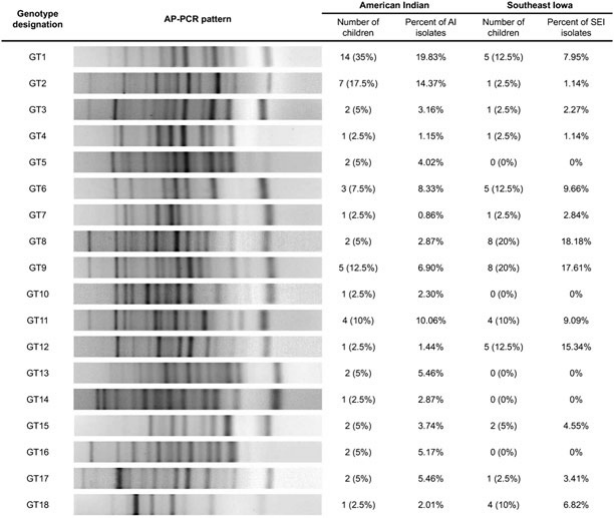
Genotypes identified with the number of children harboring each genotype reflects the diversity and commonality of *Streptococcus mutans* genotypes. The percentage of isolates within each community represents the richness of a particular genotype within the library and demonstrates the differences between the two populations

**Figure 4:**
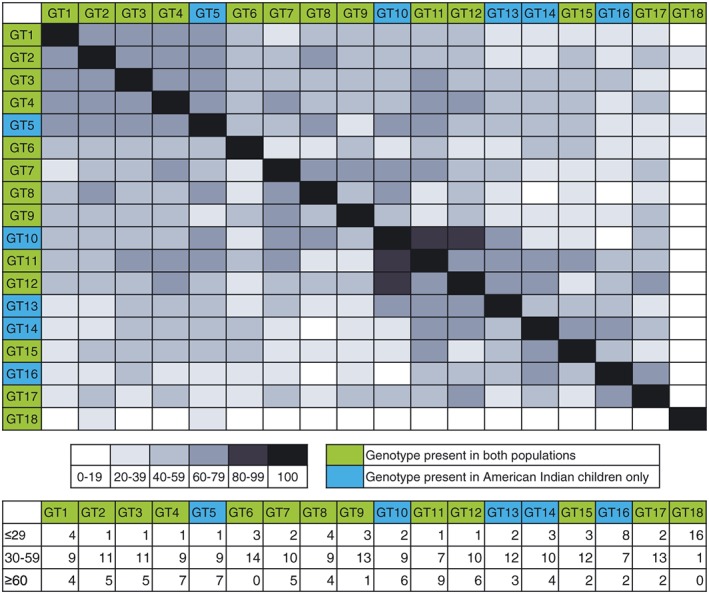
Distance matrix showing individual strain relatedness via curve‐based cluster analysis. The Pearson correlation and Unweighted Pair Group Method using Arithmetic Averages was used. Analyses were performed with GelCompar®IIv6.5 software. Numbers are expressed as percent similarities between isolates of each genotype. As this is a representative sample of one (for each genotype) instead of the full comparison, some of the similarity values are higher than in the full cluster analysis of all isolates from the subject sets, which is expected. The intent behind this smaller, single representative analysis was to see what genotypes are most distinct from the other genotypes within the comparison. The table at the bottom of the figure is a compilation of the data in the matrix showing how unique/distinct each genotype is compared to the others

A distance matrix was constructed from a curve‐based cluster analysis of one representative isolate of each unique GT utilizing the Pearson correlation and unweighted pair group method using arithmetic averages (Figure 4) to determine what GTs were most distinct from others in these populations. As this was a comparison based on a representative sample of one, some similarity values are higher than in the full cluster analysis of all isolates. Due to this expected variance, similarity values were grouped and shaded by percentages. GT16 and GT18 displayed the least similarity to other GTs. GT16 was only seen in the AI population and only isolated from two children. GT18 was observed in both populations and isolated from one AI child and four Iowa children.

## 4 DISCUSSION

Children from both populations in these studies demonstrated a limited diversity with respect to *S*. *mutans* GTs that were recovered (the mean GT recovered from all children in both population groups combined was 1.24). No child from either study harbored more than two *S*. *mutans* GTs at the time of sampling. The AI children had a greater number of children harboring two *S*. *mutans* GTs concurrently (13 children) compared to the SEI children (six children). However, the mean level of diversity within a child is consistent with other studies, as is the rarity of recovering greater than two distinct GTs from any one child at one time point (Cheon et al., 2013; Emanuelsson & Thornqvist, 2000; Klein, Flório, Pereira, Höfling, & Gonçalves, 2004; Lembo et al., 2007; Li & Caufield, 1995). Although we did not observe greater than two *S*. *mutans* GTs in any individual, we must remember that this is only one time point and it is possible and likely that these individuals could have harbored more GTs over time. In fact, the mothers of the AI children we studied sometimes harbored up to four GTs concurrently, and we did see some evidence of GT switching in the mothers and children in the AI group over time (Lynch et al., 2015). Longitudinal data are only available for the AI group, so for the purposes of this manuscript, we only considered isolates collected at the 36‐month time point in the AI children and isolates collected from one time point between 24 and 60 months (mean 42 months) in the SEI children. While a longitudinal analysis of both populations could provide clues to the stability and a deeper understanding of how the genotypic diversity of *S*. *mutans* contributes to the caries status of both populations, our cross‐sectional data do not show this. Previously published (Lynch et al., 2015) and unpublished results suggest that *S*. *mutans* colonization may not be as stable in this particular population as others have reported in other populations (Berkowitz, Jordan, & White, 1975; Köhler, Lundberg, Birkhed, & Papapanou, 2003). Additionally, reports suggest that there could be site specific differences in GT representation within an individual (Grönroos & Alaluusua, 2000). However, this study focused on similarities and differences of GTs in two populations separated both culturally and geographically. We feel that the total plaque samples, collected cross‐sectionally, are sufficient to determine the major GTs associated with each population.

We have observed large household sizes in the AI cohort. It would be interesting to compare household size and the genotypic diversity across populations. It is likely that increased household size or crowded living conditions contributes to greater horizontal colonization and therefore greater genotypic diversity within an individual (Mattos‐Graner, Li, Caufield, Duncan, & Smith, 2001).

For this study, only children that had *S*. *mutans* were analyzed from both populations. We discovered 18 distinct GTs from 348 total *S*. *mutans* isolates in the AI children and 13 GTs from 176 total *S*. *mutans* isolates in the Iowa children. Between the two populations, there were no GTs unique to the Iowa children. However, we recovered five GTs that were unique to the AI children. Moreover, there was not a great deal of commonality among the GTs found only in AI children. Therefore, it is possible that these GTs are not widely distributed in the AI population. The fact that there were approximately twice as many *S*. *mutans* isolates analyzed from the AI cohort does raise the question of whether that is the reason more GTs were detected within those subjects. While we recognize that this could potentially create the appearance of bias toward greater genotypic diversity in this population, our findings for both populations are consistent with other published studies when looking at genotypic diversity at the single subject level, as stated earlier.

This is the first study we are aware of that compares *S*. *mutans* GTs between two different populations separated by geography and ethnicity but with similar SES. However, a very recent paper compared the oral microbiome diversity among Cheyenne and Arapaho individuals in Oklahoma (Ozga, Sankaranarayanan, Tito, et al., 2016). While there was not an emphasis on *S*. *mutans* GTs, it is notable that differences between native and non‐native subjects were seen in the oral microbiomes in this cross‐sectional study. It is intriguing to find *S*. *mutans* GTs in this AI tribe that are not found in Iowa children. It is possible that *S*. *mutans* GTs unique to this population contribute to the high level of caries activity observed; however, further investigation is necessary to make that determination.

It is conceivable that there is more genetic variation or that other strep strains (i.e., *S*. *sobrinus*) could provide more genetic variation with these *S*. *mutans* GTs in the AI community. Analysis of the total plaque microbiome of these two populations may provide more information as to the diversity. Moreover, comparison of *S*. *mutans* GT profiles across additional populations is warranted and could provide interesting information on the commonality and uniqueness of specific *S*. *mutans* GTs. In both populations, no single GT comprised more than 20% of the isolates that we collected. Even GT1, which was recovered from 35% of AI children and 12.5% of Iowa children, only comprised 20% of AI isolates and 8% of Iowa isolates.

In subjects with 2 GTs present, one is dominant (making up more than 50% of that subject's recovered isolates) in 17 of 19 subjects (89.5%). Two of the SEI subjects had an equal number of isolates from each GT they displayed (no dominant GT). However, we observed no trend for GT dominance across all of the children. A dominant GT in one subject was rarely dominant in another subject. This only occurred with GT15 which was the dominant GT in two AI subjects and GT5, which was also dominant in two AI subjects; while GT12 was dominant in one AI subject and two Iowa subjects. However, of the other 12 GTs that are in individuals with multiple GTs, they were not dominant in multiple individuals. In fact, GT9 and GT18 were nondominant more often than dominant. Six of the 13 AI children with multiple GTs have GT1 as one of the GTs. It is interesting to note that while GT1 is by far the most common GT in this population, there was no instance where it was the dominant GT within an individual subject's *S*. *mutans* isolates.

Although the AI children demonstrated greater genotypic diversity, in both colonized individuals and in total isolates, it is unlikely that this could be the cause for greater caries in the AI population. It is more likely that this is the result of greater caries due to the greater likelihood of plaque dominated by *S*. *mutans* and similar acidogenic flora. Several studies have shown that greater *S*. *mutans* genotypic diversity is associated with increased tooth decay (Alaluusua, Mättö, Grönroos, et al., 1996; Napimoga, Kamiya, Rosa, et al., 2004) while other studies show the opposite effect (Kreulen, de Soet, Hogeveen, & Veerkamp, 1997), or no difference (Lembo et al., 2007). Paddick et al. demonstrated that in high‐caries individuals, genotypic diversity of commensal species, such as *Streptococcus oralis* and *Actinomyces naeslundii* is lower than that of caries free individuals (Paddick, Brailsford, Kidd, et al., 2003). It is reasonable to assume that increased genotypic diversity of *S*. *mutans* in caries active individuals could simply be a consequence of a higher *S*. *mutans* burden in these individuals and/or decreased competition from other species. A possibility remains that certain GTs exhibit specific virulence traits consistent with increased caries risk but Nascimento et al. found no such associations in their study with Brazilian adults (Nascimento, Hofling, & Goncalves, 2004). Whether certain GTs are more virulent or are associated with increased caries is the topic of ongoing research.
